# Microbial Assessment and Prevalence of Foodborne Pathogens in Natural Cheeses in Japan

**DOI:** 10.1155/2013/205801

**Published:** 2013-12-31

**Authors:** Firew Kassa Esho, Budbazar Enkhtuya, Akiko Kusumoto, Keiko Kawamoto

**Affiliations:** Section of Food Microbiology and Immunology, Research Center for Animal Hygiene and Food Safety, Obihiro University of Agriculture and Veterinary Medicine Inada-cho, Obihiro, Hokkaido 080-8555, Japan

## Abstract

The production and consumption of domestic natural cheese in Japan is increasing year by year. More than ninety percent of domestic natural cheese is produced in Hokkaido region of Japan, while information on its quality and safety related to foodborne pathogens is limited. To assess the microbiological safety of domestic natural cheese, a total of 126 natural cheese samples produced in Hokkaido were collected from December, 2012, to July, 2013. In addition to standard plate count (SPC) and coliform counts, the prevalence study of three pathogens (*Listeria monocytogenes*, pathogenic *Escherichia coli*, and *Salmonella* spp.) was performed on each sample. Real-time PCR and matrix-assisted laser desorption-ionization time-of-flight mass spectrometer methods were employed for identification of presumptive pathogens. Coliform was detected in 25 samples (19.8%) with a minimum of 25 cfu/g and a maximum of more than 3.0 × 10^6^ cfu/g. *Salmonella* spp. and *L. monocytogenes* were not isolated from any of the samples. Only one sample (0.80%) showed positive PCR amplification for *ipaH* gene suggesting possible contamination of enteroinvasive *E. coli* or *Shigella* in this product. Overall results indicate that natural cheeses produced in Hokkaido region were satisfactory microbiological quality according to existing international standards.

## 1. Introduction

Cheese consumption became popular in Japanese culinary system following the exposure of public to the western food cultures, which lead to substantial increase in domestic production and imports [1, 2]. Although the Japanese cheese market relies on imports by nearly 80%, domestic production of natural cheese is growing year by year. A recent estimate shows that over 50,000 metric tons of natural cheese is produced in Japan per year [2] out of which the majority comes from Hokkaido region. Production of natural cheese in this region accounts for more than 90% of the overall domestic production. The number of farm dairies producing natural cheese is doubled in a decade.

Consumption of cheese has been associated with foodborne outbreaks in reports from different parts of the world raising the safety concern of the product [3]. *Listeria monocytogenes* is a ubiquitous foodborne pathogen and human listeriosis outbreaks are often associated with ready-to-eat food products including cheese. Ingestion of foods contaminated with this pathogen results in a severe disease with higher fatality rate where certain risk groups including pregnant, newborn, elderly people, and immunocompromised patients are affected [4]. Several outbreaks and sporadic cases of disease associated with the consumption of pasteurized milk, cheeses made from unpasteurized milk, and other dairy products in USA and Europe in the past decades [3, 5–10]. Previous domestic studies from 1992 to 1994 reported that *L. monocytogenes* contamination was found in raw milk [11]. However, a foodborne listeriosis outbreak was occurred in 2001 due to contaminated natural cheese [12]. This is the first and only reported foodborne listeriosis in Japan so far.

In addition to *L. monocytogenes*, other enteric pathogens such as Shiga toxin-producing *Escherichia coli* (STEC) and *Salmonella *are also the causes of concern on public health in relation to the consumption of cheese worldwide [3, 5–10]. STEC is another important foodborne pathogens responsible for outbreaks which may result in hemorrhagic colitis (HC) and lethal hemolytic uremic syndrome (HUS) [13]. Although most outbreaks of HC and HUS have been attributed to serotype O157:H7, infections are also caused by other serotypes, such as O26:H11, O103:H2, O111:H8, and O145:H28 [8, 10]. *Salmonella* is another important organism which represents well-recognized foodborne bacterial pathogens. It causes a number of illnesses and deaths worldwide.

Natural cheeses, regardless of their varying characteristics, can support the growth of microorganisms including foodborne pathogens. Given the continuous increase of natural cheese consumption and following earlier reported outbreak of *L. monocytogenes* in Hokkaido associated with cheese [12], there is concern for microbiological safety of domestically produced natural cheeses. The purpose of this study was to assess a hygienic quality and safety of natural cheeses produced in Hokkaido, Japan. We performed standard plate counts (SPC) and coliform counts on each sample to obtain general hygienic information. Since a survey targeting other important pathogens in domestic natural cheese has not been performed yet, the prevalence of three significant pathogens such as *Salmonella* spp., pathogenic *E. coli, *and *L. monocytogenes* was also examined in this study.

## 2. Materials and Methods

### 2.1. Sample Collection

A total of 126 domestic natural cheeses made from raw cows' milk were collected from local retail stores and farmers' markets in Hokkaido, Japan, from December 2012 to July 2013. Some samples were obtained from online source. Sampling was centered in the Eastern Hokkaido because many natural cheese producers are located in this area, and most of cheese samples used in this study were produced on farm-dairies. The samples included soft type (*n* = 66) those were mostly ripened, semihard (*n* = 33), and hard type (*n* = 27) of cheeses. Samples were stored at refrigeration temperature (4 to 8°C) during delivery to the laboratory. Cheese type, producing companies, expiry dates, and packaging were recorded and the samples were stored in refrigerator until examined within the shelf life.

### 2.2. Bacterial Count

The homogenates of samples were prepared by aseptically removing 25 g of cheese into sterile strainer/filter-stomaching bags (Filterbag type P, GSI Creos Corporation, Tokyo, Japan). A 225 mL of buffered peptone water (BPW) was also added into the stomaching bags containing the samples and homogeneously mixed in pulsed stomacher (AES Laboratoire, Combourg, France) twice for 30 sec. The sample homogenates were serially diluted with BPW. Each serial dilution of sample homogenates was pour-plated on SPC agar (OXOID, Basingstoke, UK) and Deoxycholate agar (MERCK and Eiken Chemical, Tokyo, Japan) for SPC and coliform, respectively, followed by incubation of the plates at 37°C for 48 h.

### 2.3. Detection of Pathogens

Isolation of* L. monocytogenes* was conducted as described in International Organization for Standardization ISO 11290-1 [14]. In brief, 25 g of samples were preenriched in 225 mL of half Frazer broth (OXOID) and incubated for 24 h at 30°C. From preenriched sample, 0.1 mL of culture was enriched in 10 mL of Fraser broth (OXOID) and incubated at 35°C for 48 h. Then, a loopful of the cultures were streaked on PALCAM agar (OXOID) and incubated at 37°C for 48 h. Typical or suspect colonies were picked up, streaked on BHI agar (Becton Dickinson, NJ, USA), and incubated at 37°C for 24 h for further identification.

Isolation of *Salmonella* spp. was carried out following the procedures indicated in US Food and Drug Administration (FDA) Bacteriological Analytical Manual Online [15]. Briefly, 25 g of samples was preenriched in 225 mL buffered peptone water (MERCK) and incubated at 35°C for 18 h. Next, 0.1 mL of each sample homogenate was enriched into 10 mL of Rappaport-Vassiliadis (RV) broth (OXOID) and incubated at 42°C for 18 h. Then, a loopful of RV culture was streaked on deoxycholate hydrogen sulfide lactose agar (DHL; Eiken Chemical) and CHROMagar *Salmonella* and incubated at 37°C for 24 h. Suspicious colonies were collected and streaked on BHI agar for further analysis by Matrix-assisted laser desorption-ionization time-of-flight mass spectrometer (MALDI-TOF MS).

Screening for pathogenic *E. coli* (STEC, enterotoxigenic *E. coli*, enteropathogenic *E. coli*, enteroaggregative *E. coli*, and enteroinvasive *E. coli*) was performed by real-time PCR based on their associated genetic markers. Briefly, 25 g of samples was preenriched in 225 mL of mEC broth with novobiocin (MERCK) and incubated at 37°C for 24 h. DNA was extracted from 2 mL of preenriched broth by using PrepMan Ultra Sample Preparation kit (Applied Biosystems, Foster City, CA, USA). Real-time PCR was performed using QuickPrimer kit (*stx 1*, *stx 2*, *ipaH*,* LT*,* EASTI*,* STI*) (Takara Bio, Shiga, Japan) according to the manufacturer's instruction. QuickPrimer Control DNA sets were used as positive control while DDW was used as negative control of the DNA amplification.

### 2.4. Identification of Bacteria by MALDI-TOF MS

MALDI-TOF MS analysis was used to identify presumptive isolates. Bacterial cells of single colony grown on BHI agar plates were transferred to a 96 well stainless steel target plate (Bruker Daltonik, Germany) using a disposable loop. The sample on the plate was then overlaid with 1 *μ*L of *α*-Cyano-4-hydroxycinnamic acid and allowed to dry at room temperature. The plate was then subjected to MALDI-TOF Mass Spectrometer machine (autoflex-04S, Bruker Daltonik) and profile spectra were analyzed using MALDI Biotyper 2.0 software (Bruker Daltonik) according to the reference database.

## 3. Result

Amongst the total samples inspected (126 samples), 66, 33, and 27 of them were soft, semihard, and hard type of cheeses, respectively. All natural cheese samples in this study was already precut and prepacked individually at the time of purchase. Soft cheeses tested (66 samples) include brie, camembert, cream cheese, gorgonzola, mozzarella, and wash-type soft cheeses. Semi-hard (33 samples) and hard type cheese (27 samples) samples include cheddar, caciocavallo, emmental, gouda gruyere, and raclette.

**Figure 1:**
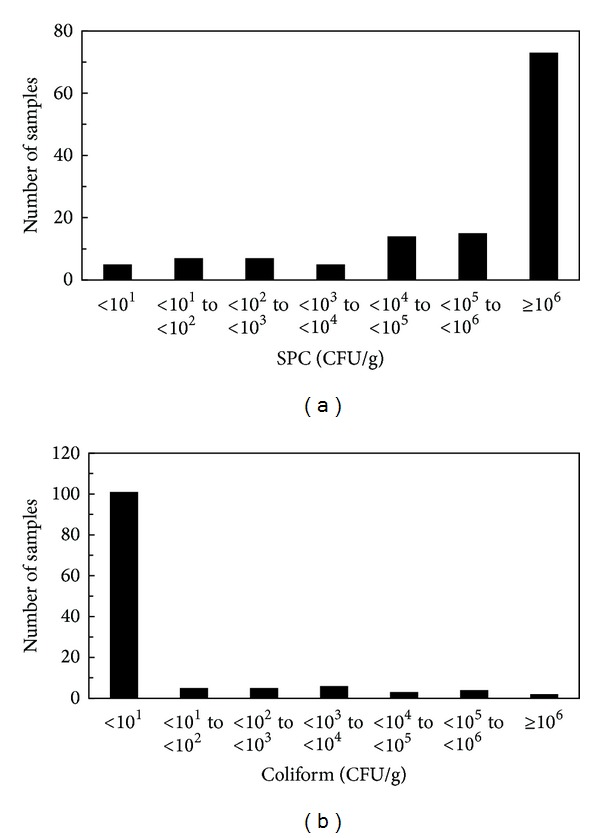
Hygienic quality of natural cheese samples. Distribution of SPC (a) and coliform counts (b) in natural cheese samples.

SPC of natural cheese tested in this study ranged from below detection limit (<10 cfu/g) to 6.4 × 10^6^ CFU/g (Figure 1(a)). Five (4.0%) out of 126 samples were negative (<10 cfu/g) for viable aerobic bacteria. SPC counts are generally used for monitoring microbial quality and spoilage levels. However, fermented products like cheese generally show high number of SPC because “good” microorganisms present in food to ferment properly. High level of SPC in natural cheese samples seems to be natural, since some lactic bacteria and mold are known to grow on SPC agar.

No coliforms were detected in 80.2% of samples tested in this study, and 25 of cheese samples (19.8%) were found to be positive for coliform bacteria (Table 1). Coliform counts ranged from below detection limit (<10 cfu/g) to over detection limit (>3.0 × 10^6^ CFU/g) (Figure 1(b)).

Soft type cheese showed the highest positivity of coliform compared to other cheese types. Out of the total 66 soft cheese samples, 18 samples (27.3%) were positive for coliform with minimum and maximum values of 2.8 × 10^1^ and more than 3.0 × 10^6^ CFU/g, respectively. On the other hand, from the 33 semi-hard types of cheese samples, 9.1% of them were positive for coliform with minimum and maximum values of 8.0 × 10^2^ and 8.0 × 10^5^ CFU/g, respectively. Among 27 samples of hard type cheese, four samples (14.8%) were positive for coliform count with minimum and maximum values of 2.5 × 10^1^ and 2.7 × 10^4^ CFU/g, respectively.

We further classified the type of packaging into 7 types such as AF/other (primary packaging: aluminum foil wrapping, secondary: none, plastic bag or wooden box), Paper A (paper wrapping only), Paper B (primary: paper, secondary: carbon box, wooden box or plastic bag), Plastic A (plastic vacuum seal), Plastic B (plastic film wrapping), Plastic C (plastic container), and Can (canned). Table 1 shows the relatedness between coliform contamination and cheese and packaging types. As shown in this Table, various packagings were applied for soft cheeses, whereas most of semi-hard and hard type cheeses were plastic vacuum sealed. Soft type cheeses wrapped with paper (Paper A in Table 1) showed the highest coliform-positive rate such as 100.0%. Although the number of samples were as low as 5, this is a notable difference compared to the other packagings. The prevalence of coliform in soft cheeses that were paper-wrapped but also employed secondary outer packaging such as carbon box, wooden box, or plastic bags (Paper B in Table 1) showed lower contamination. Coliform-positive rate of plastic packaging including film wrapping (25.0%), vacuum seal (18.8%), and container (0.0%) were much lower compared to paper wrapping.

For detection of pathogens, we used standard analytical methods of ISO and FDA as well as Japanese method of Ministry of Health, Labor and Welfare (MHLW). For identification of presumptive isolates, we used MALDI-TOF MS analysis and real-time PCR analysis. The result showed that among the 126 samples tested for *L. monocytogenes* none of them was positive for this pathogen. Likewise, no *Salmonella* spp. was detected from 126 natural cheese samples. However, under real-time PCR inspection, none of the 126 samples inspected was positive for pathogenic *E. coli*, except one sample (0.79%) that showed positive result for *ipaH *gene, indicating possible presence of EIEC or *Shigella* spp. [16]. However, we failed to isolate the pathogen from the cheese sample by culture-based detection method using specific media for *Shigella* spp.

## 4. Discussion

Despite the growing popularity of domestic natural cheeses among consumers, information about microbiological safety of these products is limited. The incidence of high coliform count in food is considered as indicator for reduced hygienic condition in production process. The results of this study shows that about 80.2% of the 126 natural cheese samples examined were negative for coliforms. Although microbiological criteria for *L. monocytogenes* in natural cheese (negative per 25 g) are described by the Ministry of Health, Labour and Welfare, other criteria, for example, coliform count, *E. coli*, and the other pathogenic bacteria, are not specified. However, it is a prerequisite to use pasteurized milk for natural cheese production in Japan. In case of using unpasteurized raw milk, producers must process products under equivalent temperature condition (63°C for 30 min) to eliminate the risk of pathogenic organisms (Ministry of Health, Labour and Welfare, 2003). Thus, coliform bacteria are not expected to exist due to the heating process in the production of natural cheese. Although milk pasteurization is regarded as an effective method to eliminate bacteria including foodborne pathogens, it is suggested that postpasteurization contamination and poor hygienic practices such as inappropriate pasteurization of raw milk and equipment also one of the causative factor in cheese-related foodborne illness [17].

The present study revealed significant correlation between packaging type and rate of coliform contamination in soft type cheese as a results of their packaging in paper with or without of secondary packaging (31.8% and 100.0% resp.), plastic packaging by film wrapping, vacuum or container (25.0%, 18/8% and 0.0% resp.), aluminum foil (20.0%) and canned (0.0%). The microbiological quality of natural cheese is influenced by equipment and environmental hygiene during production, packaging and handling and storage conditions as well as by the quality of raw milk. It was not determined in this study whether the highest coliform prevalence in paper-wrapped cheese is due to pre-packaging or post-packaging. However, our results clearly indicate that appropriate packaging such as plastic packaging and/or combination of primary and secondary packaging will reduce the risk of contamination. To choose an appropriate packaging that is suitable for the products is a simple and practical measure to reduce the risk of unnecessary contamination and ensure the food safety. We are planning to discuss about our data with the relevant cheese manufacturers whose products were observed in the high levels or continuous contamination of coliform during the survey for making a good hygiene practice plan and appropriate HACCP plan, by collecting and analyzing the data of coliform contamination levels in pre-and post production process including raw milk, pasteurization, factory environment such as utensils and equipment, storage and ripened shelf, and packaging materials, and so forth.

Most of the natural cheese samples tested in this study complied with the microbiological criteria by Hokkaido regional accreditation body, which is the food standard (criteria) uniquely given by Hokkaido Government. Since Hokkaido prefecture is the nation's first and largest area in the production of a wide array of agricultural, seafood and fresh dairy products including natural cheese (Spotlight on Hokkaido, NatureJobs article in 2011), ensuring food safety of the food products those made in Hokkaido region is the one of the important issues. Hokkaido regional accreditation requires natural cheese to be negative for coliform and *L. monocytogenes *in 25 g by the standard microbiological test (http://www.pref.hokkaido.lg.jp/ns/shs/06/ninshou/cheese-kijyun.pdf). Similarly, the natural cheese samples tested in this study were within the limit of US ordinance for pasteurized grade “A” milk which states coliform should be lower than 10 cfu/mL [18]. Applying criteria in the European standard that requires coliform in cheese produced from heat-treated milk to be less than or equal to 10^5^ cfu/g [16], 95.2% of samples inspected in this study were within this limit.

The prevalence of foodborne pathogens including *L. monocytogenes*, pathogenic *E. coli* (STEC, ETEC, EPEC, EAEC, and EIEC), and *Salmonella* spp. in natural cheese was investigated in this study. *L. monocytogenes*,STEC, and *Salmonella *are reportedly associated with foodborne outbreaks related to the consumption of cheese [3, 5–10]. Apart from the single one, all the other samples inspected had no prevalence of the foodborne pathogens such as *L. monocytogenes*, *Salmonella* spp., and pathogenic *E. coli*. The results of the present study also demonstrate that natural cheeses produced in Hokkaido are negative for these relatively frequent foodborne pathogens of the recent times. The *ipaH* gene detected from one sample (0.79%) in this survey indicates the possibility of contamination by EIEC or *Shigella*, because this gene is a multicopy gene which is exclusively found in those pathogens [19]. The source of contamination is uncertain, but fecally contaminated water and unsanitary handling by food handlers are the probable causes of contamination. Previous report of *S. sonnei* outbreak in Spain was associated with regionally manufactured fresh pasteurized milk cheese that caused large outbreak affecting over 200 people in the region [20]. Epidemiological investigation suggests that infected employee at the cheese factory might have been the source of contamination.

Several prevalence studies conducted across the globe on these pathogens in natural cheeses show varying results from place to place. No pathogens such as *E. coli* O157:H7, *L. monocytogenes*, *Salmonella,* or *Campylobacter* was detected in US study [21]. In UK study, *Salmonella* was not detected; however, 2% of samples were at unsatisfactory level of *Staphylococcus aureus*,* E. coli*, and *L. monocytogenes*. Study in Italy showed that no *L. monocytogenes* was detected; however, high levels of *S. aureus* and *E. coli* were detected in particular samples [22]. In Spanish study, 2.4% of samples were positive for STEC [23]. In Peru, 7.8% of samples were positive for *E. coli* O157:H7 [23, 24]. From our results and the fact that outbreaks of food borne pathogens related to consumption of cheese are not common in Japan, natural cheese produced in Hokkaido seems to be in relatively better hygienic status as compared to those produced in other countries mentioned above.

The outbreaks of food borne pathogens related to consumption of dairy products are not common in Japan so far. Several reports predict that the market for domestically produced natural cheese will continue to grow from year to year [1, 2]. The average cheese consumption of the Japanese is about 2 kg a year per person, which is 10 times lower than those in European countries. This may account for lower incidence of foodborne outbreak associated with cheese. However, increase in consumption and production amounts may influence the risk of foodborne illness in future. Conducting similar survey in small and large scale on periodic basis would be important to ensure food safety of domestic natural cheese and to prevent possible incidence of foodborne pathogen outbreaks and related public health hazards.

In conclusion, the current result indicates that the microbiological quality and hygienic status of the natural cheese tested in this study was in the mostly fine and satisfactory status. This may be attributed to the required use of pasteurized milk for production of dairy products and implementation of good manufacturing and hygienic practices across all production stages from farm to table. The results from this study also suggest that current regulation and ministerial guidance would be considered appropriate for safe natural cheese production.

## Figures and Tables

**Table 1:** Coliform-positive rates in the different types of cheese and packaging.

Cheese types	Package types (% of coliform positive)							Total
	AF/other^a^	Paper A^b^	Paper B^c^	Plastic A^d^	Plastic B^e^	Plastic C^f^	Can	
Soft	1/5 (20.0)	5/5 (100.0)	7/22 (31.8)	3/16 (18.8)	2/8 (25.0)	0/9 (0.0)	0/1 (0.0)	**18/66 (27.3)**
Semi-hard	0	0	0	3/32 (9.4)	0/1 (0)	0	0	**3/33 (9.1)**
Hard	0	0	0	4/27 (14.8)	0	0	0	**4/27 (14.8)**
Total	**1/5 (20.0)**	**5/5 (100.0)**	**7/22 (31.8)**	**10/75 (13.3)**	**2/9 (22.2)**	**0/9 (0.0)**	**0/1 (0.0)**	**25/126 (19.8)**

^a^Primary packaging: aluminum foil wrap, secondary: none, plastic bag or wooden box.

^b^Paper wrapping only.

^c^Primary packaging: paper, secondary: carbon box, wooden box or plastic bag.

^d^Plastic vacuum seal.

^e^Plastic film wrapping.

^f^Plastic container.
